# Knee flexion contracture impacts functional mobility in children with cerebral palsy with various degree of involvement: a cross-sectional register study of 2,838 individuals

**DOI:** 10.1080/17453674.2021.1912941

**Published:** 2021-04-18

**Authors:** Evelina Hanna Sofia Pantzar-Castilla, Per Wretenberg, Jacques Riad

**Affiliations:** a Department of Orthopedic Sugery, Örebro University Hospital; b Department of Orthopedics, Örebro University; c Department of Orthopaedics, Skaraborg Hospital, Skövde, Sweden

## Abstract

Background and purpose — The impact of knee flexion contracture (KFC) on function in cerebral palsy (CP) is not clear. We studied KFC, functional mobility, and their association in children with CP.

Subjects and methods — From the Swedish national CP register, 2,838 children were defined into 3 groups: no (≤ 4°), mild (5–14°), and severe (≥ 15°) KFC on physical examination. The Functional Mobility Scale (FMS) levels were categorized: using wheelchair (level 1), using assistive devices (level 2–4), walking independently (level 5–6). Standing and transfer ability and Gross Motor Function Classification (GMFCS) were assessed.

Results — Of the 2,838 children, 73% had no, 14% mild, and 13% severe KFC. KFC increased from 7% at GMFCS level I to 71% at level V. FMS assessment (n = 2,838) revealed around 2/3 were walking independently and 1/3 used a wheelchair. With mild KFC (no KFC as reference), the odds ratio for FMS level 1 versus FMS level 5–6 at distances of 5, 50, and 500 meters, was 9, 9, and 8 respectively. Correspondingly, with severe KFC, the odds ratio was 170, 260, and 217. In no, mild, and severe KFC 14%, 47%, and 77% could stand with support and 11%, 25%, and 33% could transfer with support.

Interpretation — Knee flexion contracture is common in children with CP and the severity of KFC impacts function. The proportion of children with KFC rose with increased GMFCS level, reduced functional mobility, and decreased standing and transfer ability. Therefore, early identification and adequate treatment of progressive KFC is important.

Knee flexion contracture is a common problem in children with cerebral palsy (CP) (Miller 2005, Cloodt et al. 2018). Due to muscle imbalance, short and spastic hamstring muscles, and prolonged sitting posture, knee flexion contracture may develop and often progresses in adolescence (Miller 2005, Rodda et al. 2006). Although the exact impact of knee flexion contracture and its contribution to the development of flexed knee gait is still not fully understood, it is associated with progressive deterioration of gait in the ambulating child (Bell et al. 2002, Rodda et al. 2006) and it results in difficulties maintaining functional standing, sitting, and transfer in non-ambulatory children (Miller 2005, Cloodt et al. 2018). In addition, knee flexion contracture generates increased forces on the knee joint, which may cause pain (Rodda et al. 2006, Steele et al. 2012, Schmidt et al. 2020).

Prevention of knee flexion contracture has not been thoroughly studied, and physiotherapy treatment and focal spasticity reduction have been attempted without convincing effect (Hägglund et al. 2005, Galey et al. 2017). In ambulatory children, there are several reports of improvement of gait pattern and knee flexion contracture after orthopedic surgery (Ma et al. 2006, Rodda et al. 2006, Stout et al. 2008, Taylor et al. 2016). These studies are limited mainly to children in Gross Motor Function Classification System (GMFCS) level I–III, and occasionally level IV, and varies across age groups as well as according to the surgery performed (Ma et al. 2006, Rodda et al. 2006, Stout et al. 2008, Taylor et al. 2016).

The Functional Mobility Scale (FMS), the Pediatric Outcomes Data Collection Instrument (PODCI), and the Gross Motor Function Measure dimension D (GMFM D) are often used to assess function after orthopedic surgery; all three instruments describe how the child actually moves in daily life, and not necessarily what his or her capacity is (Russell 1993, Daltroy et al. 1998, Graham et al. 2004).

Knee flexion contracture is easy to assess by physical examination; however, there are limited reports on the prevalence of knee flexion contracture and distribution of functional mobility in larger cohorts of children with CP at all GMFCS levels (Rodby-Bousquet and Hägglund 2010, Cloodt et al. 2018). We studied knee flexion contracture, functional mobility, and their association in children with CP. We assumed that the presence and severity of knee flexion contracture contributes to decreased physical function in children with CP.

## Subjects and methods

This cross-sectional study was based on data from the Swedish national Cerebral Palsy register (www.cpup.se). More than 95% of all children with CP in Sweden born from 2000 onwards are included in the CPUP register and participate in the follow-up program, which includes a physical examination and assessment of function by their local physiotherapist yearly or every other year (Alriksson-Schmidt et al. 2017). All children in Sweden with CP are enlisted in local “habilitation” centers, where specially trained physiotherapists perform the physical examination including passive range of motion and use the same instructions to assess physical functional including the Functional Mobility Scale (FMS) and Standing and Transfer ability (Assessment form physiotherapist, www.cpup.se).

**Figure 1.**
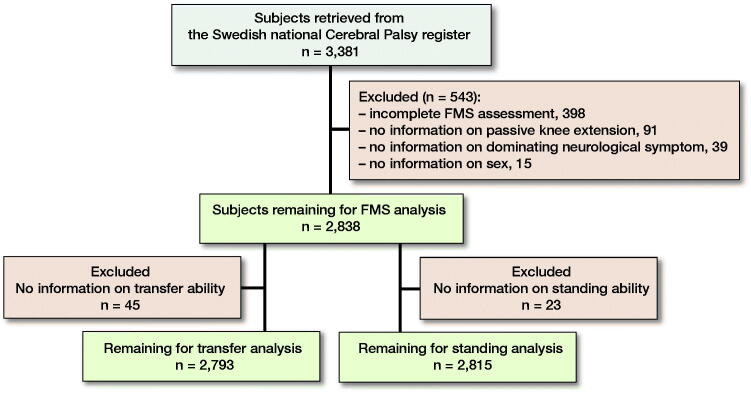
Flow chart of inclusion.

We identified children born between 1999 and 2016 and retrieved data from their most recent assessments, 2017 and 2018. The inclusion criteria were children with CP and age range 4–18 years. The exclusion criteria were missing data in the register for sex, dominating neurological symptom, passive range of motion of the knee joint, and mobility. After exclusions, 2,838 remained for FMS analysis, 2,793 for transfer analysis, and 2,815 for standing analysis (Figure 1). The degree, classification, and distribution of knee flexion contracture, FMS, standing, and transfer ability ratings were collected for all children with data for each measure.

### Knee flexion contracture

In the physical examination, passive knee extension was measured in a standardized way with a goniometer on the lateral side of the thigh and shank in supine position, with the hip in full extension and the ankle in plantar flexion. For this study, we defined knee flexion contracture of 5–14° as mild and 15° or more as severe, based on the levels used in the CPUP and by Cloodt et al. (2018). We classified knee flexion contracture of 4° or less as “no.” We used the data from the leg with the least knee extension (most knee flexion contracture) for analysis.

### Functional Mobility Scale (FMS)

The FMS is a validated evaluation measure of functional mobility in children with CP between 4 and 18 years of age (Graham et al. 2004). The purpose of FMS is to classify the individual’s present and predominant functional mobility at 3 specific distances, 5, 50, and 500 meters, corresponding to at home, in school, and out in the wider community, respectively. The FMS classification is reached through questions posed to the child and parents. It consists of a 6-level ordinal scale, indicating the child’s level of mobility independence:

FMS level 1 = uses wheelchair; 2 = uses a walker; 3 = uses crutches; 4 = uses 1 or 2 canes; 5 = walks independently on even surfaces; 6 = walks independently on all surfaces.

For the purpose of this study, we grouped the FMS levels according to use of mobility aids and assistive devices: FMS level 1, FMS level 2–4, and FMS level 5–6. The same grouping was used for all 3 distances, 5, 50, and 500 meters.

### Standing and transfer ability

Standing and transfer ability assessments are included in the CPUP follow-up program and mainly derive from the International Classification of Functioning, Disability and Health (WHO). The physiotherapist asks how the child usually performs standing (categorized as without support, with support, or cannot stand) and transfer from sitting to standing and from standing to sitting (categorized as without support, with support, or cannot transfer).

### Statistics

Multinomial regression was used to analyze the categorical outcome FMS distances (5, 50, and 500 meters), with level 5–6 (walking independently) as the outcome reference. The independent variable was knee flexion contracture categorized as: no (≤ 4°, mild (5–14°), severe (≥ 15°), and the analysis was adjusted for age and sex. The categorical outcome “standing” with category “standing without support” as reference and the outcome transfer “ability” with category “transferring without support” as reference was analyzed in the same way. The regression model is based on the assumption that the presence and severity of KFC contributes to decreased physical function. A multinomial regression gives odds ratio (OR) with 95% confidence interval (CI) as association measures. A p-value of less than 0.05 was regarded as statistically significant. All statistical analyses were performed with SPSS, version 22 (IBM Corp, Armonk, NY, USA).

### Ethics, data sharing, funding, and potential conflicts of interest

The Research Ethics Committee at Lund University, Sweden, approved the study, and permission to extract data was obtained from the registry holder. The datasets used and analyzed during the current study are available from the corresponding author on reasonable request.

This research did not receive any specific grant from funding agencies. The authors declare that they have no conflict of interest.

## Results

### Demographics

This study included 2,838 children between 4 and 18 years, mean 10 years 7 months (SD 3.9). The distribution of age, sex, and GMFCS levels is presented in Table 1. The dominating symptom was spastic CP, reported in 79%; dyskinesia in 11%; and ataxia in 5%. The remaining 5% had a mixed symptom pattern.

**Table 1.** Distribution of age, sex and Gross Motor Function Classification System (GMFCS) level (n = 2,838)

				GMFCS level				
Age	n	Male (58%)	Female (42%)	I (50%)	II (17%)	III (8%)	IV (11%)	V (14%)
4–6	580	332	248	323	86	35	58	78
7–9	601	360	241	283	120	46	65	87
10–12	696	414	282	367	121	56	69	83
13–15	583	338	245	266	96	51	84	86
16–18	378	200	178	169	64	42	48	55
Total	2,838	1,644	1,194	1,408	487	230	324	389

### Knee flexion contracture

No knee flexion contracture (≤ 4°) was noted in 73% of the 2,838 children, mean 2° (SD 4°, range –35° to 4°). Mild knee flexion contracture was present in 14%, mean 7° (SD 3°, range 5°–13°), and 13% had severe knee flexion contracture, mean 27° (SD 12°, range 15°–95°).

In the age group 4–6 years, 13% had mild or severe knee flexion contracture (≥ 5°). The proportion rose to 20% in the age group 7–9 years, 27% in the age group 10–12, and 40% in each of the older age groups (13–15 and 16–18 years). Similarly, the proportion of knee flexion contracture rose with increased GMFCS level (Table 2).

**Table 2.** Distribution of knee flexion contracture (no, mild, severe) in relation to GMFCS levels. Values are count (%)

	Knee flexion contracture		
GMFCS level	No ≤ 4°	Mild 5–14°	Severe ≥ 15°
I	1,310 (93)	95 (6.7)	3 (0.3)
II	402 (83)	74 (15)	11 (2)
III	123 (54)	62 (27)	45 (20)
IV	126 (39)	83 (26)	115 (35)
V	113 (29)	89 (23)	187 (48)

### Functional Mobility Scale

**Figure 2.**
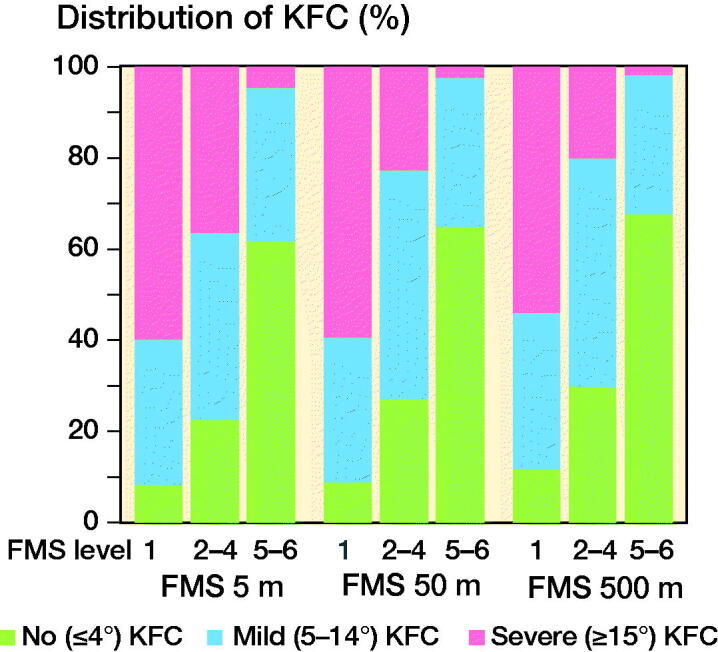
Distribution of Functional Mobility Scale (FMS) in no, mild, and severe knee flexion contracture (KFC). FMS level 1 (using wheelchair), 2–4 (using assistive devices), and 5–6 (walking independently), (n = 2,838).

Lower FMS levels were noted with increased knee flexion contracture (Table 3, Figure 2).

**Table 3.** Distribution of knee flexion contracture (no, mild, severe) in relation to the Functional Mobility Scale (FMS) level 1 (using wheelchair), levels 2–4 (using assistive devices), and levels 5–6 (walking independently), and standing and transfer ability (cannot, with support, and without support). Values are count (%)

	Knee flexion contracture		
Factor	No ≤ 4°	Mild 5–14°	Severe ≥ 15°
FMS 5 meters			
FMS level 1	252 (12)	183 (46)	312 (86)
FMS level 2–4	93 (5)	38 (9)	29 (8)
FMS level 5–6	1,729 (83)	182 (45)	20 (6)
FMS 50 meters			
FMS level 1	282 (14)	198 (49)	333 (92)
FMS level 2–4	128 (6)	43 (11)	17 (5)
FMS level 5–6	1,664 (80)	162 (40)	11 (3)
FMS 500 meters			
FMS level 1	440 (21)	244 (61)	345 (96)
FMS level 2–4	69 (3)	21 (5)	8 (2)
FMS level 5–6	1,565 (76)	138 (34)	8 (2)
Standing ability			
Cannot	18 (1)	18 (5)	60 (17)
With support	282 (14)	191 (47)	274 (77)
Without support	1,757 (85)	192 (48)	23 (6)
Transfer ability			
Cannot	144 (7)	117 (29)	220 (62)
With support	214 (11)	97 (25)	114 (33)
Without support	1,681 (82)	183 (46)	15 (5)

### Standing and transfer ability

**Figure 3.**
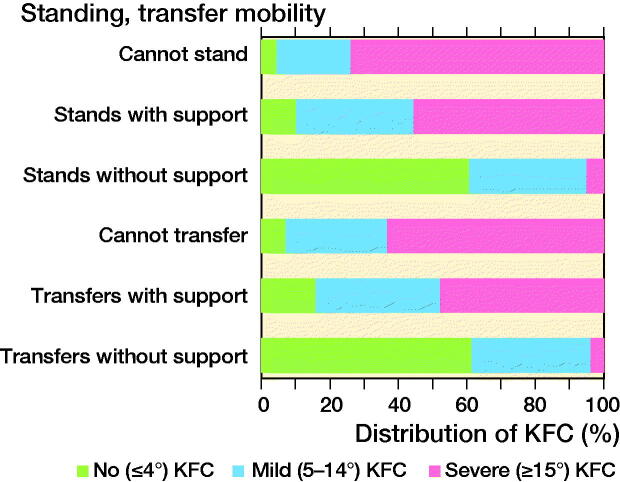
Distribution of standing (n = 2,815) and transfer Ω (n = 2,793) ability in no, mild, and severe knee flexion contracture (KFC). Based on the International Classification of Functioning, Disability and Health (ICF). Standing refers to the child’s ability to maintain a basic body position when standing. Transfer mobility refers to the child’s ability to transfer from sitting to standing and from standing to sitting.

The proportion of children with decreased standing and transfer ability rose with increased knee flexion contracture (Table 3, Figure 3).

### Association between knee flexion contracture and FMS

The odds ratios (OR) with 95% confidence interval (CI) are adjusted for age and sex. In children with mild knee flexion contracture, for both FMS 5 and 50 meters, we found an OR of 9 (CI 7–11) between FMS level 1 and level 5–6, while the OR was 4 (3–6) between FMS level 2–4 and level 5–6. For FMS 500 meters, the corresponding ORs were 8 (6–10) and 4 (2–6), respectively (Table 4).

**Table 4.** Multinomial regression with outcome Functional Mobility Scale level for distances of 5, 50, and 500 meters and independent variable knee flexion contracture (no, mild, and severe)

Distance Knee flexion contracture	FMS level 1 versus 5–6		FMS level 2–4 versus 5–6	
	OR (95% CI)	p-value	OR (95% CI)	p-value
No (≤ 4°)	Reference		Reference	
FMS 5 m				
Mild (5–14°)	9 (7–11)	< 0.001	4 (3–6)	< 0.001
Severe (≥ 15°)	170 (103–279)	< 0.001	32 (17–61)	< 0.001
FMS 50 m				
Mild (5–14°)	9 (7–11)	< 0.001	4 (3–5)	< 0.001
Severe (≥ 15°)	266 (141–500)	< 0.001	22 (10–49)	< 0.001
FMS 500 meters				
Mild (5–14°)	8 (6–10)	< 0.001	4 (2–6)	< 0.001
Severe (≥ 15°)	217 (161–445)	< 0.001	24 (9–66)	< 0.001

The analysis was adjusted for age and gender. FMS = Functional Mobility Scale, OR = odds ratio, CI = confidence interval.

In children with severe knee flexion contracture, for FMS 5 meters, the OR was 170 (103–279) between FMS level 1 and level 5–6, and 32 (17–61) between FMS level 2–4 and level 5–6. For FMS 50 meters, the corresponding ORs were 266 (141–500) and 22 (10–49), respectively. For FMS 500 meters, the corresponding ORs were 217 (161–445) and 24 (9–66) (Table 4).

### Association between knee flexion contracture and standing ability

In children with mild knee flexion contracture, we found an OR of 12 (6–23) between being unable to stand and standing without support, and an OR of 8 (6–10) between standing with support and standing without support. In children with severe knee flexion contracture, the corresponding ORs were 388 (186–806) and 113 (71–180), respectively.

### Association between knee flexion contracture and transfer ability

Concerning the ability to transfer from sitting to standing and from standing to sitting, in children with mild knee flexion contracture we found an OR of 10 (7–13) between being unable to transfer and transferring without support, and an OR of 5 (4–6) between transferring with support and transferring without support. In children with severe knee flexion contracture, the corresponding ORs were 315 (176–565) and 77 (43–137), respectively.

## Discussion

In our study with 2,838 children at all GMFCS levels, 73% had no, 14% mild, and 13% severe knee flexion contracture. Functional mobility assessment revealed, when analyzing the total cohort, that around 2/3 were walking independently and 1/3 used a wheelchair. Most children could stand and transfer from sitting to standing without support. We found strong associations between both mild and severe knee flexion contracture and functional mobility, standing, and transfer ability.

### Knee flexion contracture

There is no widely adopted definition of mild, moderate, and severe knee flexion contracture in CP. Miller (2005) defined moderate knee flexion contracture as from 10–30° and severe knee flexion contracture as over 30°. Ma et al. (2006) reported 3 different treatment approaches depending on the severity of knee flexion contracture: 10–15° 15–25°, and over 25°. Cloodt et al. (2018) reported 22% knee flexion contracture of 5°or more, including all GMFCS levels, which is similar to the 27% found in our study; however, their study did not include any functional measures besides GMFCS. In line with previous studies, we noted that knee flexion contracture increases with age and GMFCS level (Hägglund and Wagner 2008, Nordmark et al. 2009, Cloodt 2018).

### Functional Mobility Scale

Harvey et al. (2010) reported substantial agreement between FMS ratings and direct observation, demonstrating the validity of FMS as a measure of performance for children with CP. In post-surgery rehabilitation, the FMS has proven sensitive enough to detect both initial deterioration and ultimate improvement in mobility (Graham et al. 2004, Harvey et al. 2007). Around 2/3 of all children were high functioning, walking independently (FMS level 5–6) at the 3 distances 5, 50, and 500 meters. Around 1/3 used a wheelchair (FMS level 1) for the 3 distances. Only a small percentage of the children move with assistive devices (FMS level 2–4), which is similar to the results by Rodby-Bousquet and Hägglund (2012) in a study of 562 children. Even though many children with CP have the capacity to walk with assistive devices, it may not be how they actually move in daily life (Graham et al. 2004, Palisano et al. 2009, Wilson et al. 2014). Wilson et al. (2014) found that, even though walking capacity in a 1- and 5-minute walking test was associated with FMS level in children at GMFCS levels I to III, much of the variance found remained unexplained. Palisano et al. (2009) reported that young people with CP, although capable of walking in the community environment, chose mobility solutions that were faster and more efficient. The social aspect of being able to keep up with their peers could also be one reason for the finding that few children were in FMS level 2–4 in our study. It has also been reported that pain in children with CP was primarily explained by bone and joint deformities, including knee flexion contracture, and additionally associated with reduced mobility, as evaluated with the FMS (Schmidt et al. 2020). Another explanation could be economic factors: in Sweden, the assistive technology centers, at no cost to the family, provide assistive devices and aids such as wheelchairs. Our results are difficult to compare with the original study on FMS classification by Graham et al. (2004), since their study group consisted of 310 children from a tertiary referral center, in contrast to our population-based group of 2,838 children.

### Standing and transfer ability

Rodby-Bousquet and Hägglund (2010), in a study of 562 children from the CPUP register, reported similar results to ours on standing and transfer ability. They concluded that the GMFCS scale is useful for prediction of the individual child’s future sitting and standing performance. However, their study did not include any data on knee flexion contracture or other deformities in the lower limb.

### Knee flexion contracture and physical function

Ma et al. (2006) noted improvements in knee flexion contracture, gait kinematics, and FMS level for 5 and 50 meters after hamstring lengthening and transfer; furthermore, they noted an association between knee flexion contracture and FMS, which is in line with our results. Taylor et al. (2016) showed that children with CP (mainly those at GMFCS levels I–III) and crouch gait who underwent single-event multilevel surgery (SEMLS), including posterior knee capsulotomy or distal femur extension osteotomy, improved in passive knee extension on physical examination and knee kinematics in gait. However, they observed no improvement in the functional measure GMFM D, despite long-term follow-up. This suggests that the FMS could be more sensitive for detecting changes than GMFM D.

Furthermore Ammann-Reiffer et al. (2019) reported that a change of 1 FMS level is a clinically meaningful change in the rehabilitation of gait performance in children with motor disorders, though not exclusively CP. The Gillette Functional Assessment Questionnaire has proven sensitive and detected improved function after surgery (Novacheck et al. 2000), but we did not use it in our study. Harvey et al. (2007) pointed out that FMS level decreases initially after SEMLS and then improves 24 months postoperatively. Interestingly, Harvey et al. showed that the FMS was able to detect changes over time postoperatively for children at GMFCS level III, whereas the GMFCS remained stable. This is in line with the observation by Palisano et al. (1997) that GMFCS is stable over time and does not respond to change after interventions.

Knee flexion contracture is often present in children with crouch gait, with increased knee flexion throughout stance phase, and not as common in jump gait pattern where increased knee flexion is mainly noted in early stance (Sutherland and Davids 1993, Rodda et al. 2004). Knee extensor mechanism insufficiency may add dynamic knee flexion in stance to the static knee flexion contracture found on physical examination.

In the surgical treatment of flexed knee gait the correction of rotational malalignment with increased internal rotation of the femur and external tibial torsion, and foot deformity, should be included. The common deformity pes planovalgus with midfoot break results in an unstable foot with a short lever arm and deviation of foot progression. A stable plantigrade foot with forward progression is important for both gait and standing, providing a long lever arm for the ground reaction force to act on, to develop a sufficient plantar flexion knee extension couple moment, extending the knee (Miller 2005, Young et al. 2010).

Young et al. (2010) stated that in children with CP, over time, knee flexion moments develop and increase the forces driving towards flexed knee gait and knee flexion contracture. In our study, we assumed that the severity of knee flexion contracture impacts physical function. In crouch gait, besides GMFCS level, severe knee flexion contracture plays a major role and surgical treatment with distal femoral extension osteotomies and patellar tendon advancement is often warranted (Stout et al. 2008). In contrast to mild knee flexion contracture in GMFCS level I and II, the role of knee flexion contracture is not as obvious (Ma et al. 2006, Young et al. 2010). The flexed knee gait seen in younger children is ideally treated before knee flexion contracture develops, and the timing and dose of treatment is an important factor (Miller 2005, Young et al. 2010).

Our results, showing a clear association between both mild and severe knee flexion contracture and FMS, standing, and transfer ability, support the usefulness of these functional assessments for all GMFCS levels. It is not only the presence of KFC that affects physical function but also the severity. KFC is more frequently noted with higher GMFCS levels, which is also true regarding our main outcome variables, lower FMS levels, decreased standing, and transfer ability. Furthermore, the FMS has also been used to study how the care provided during childhood impacts CP in adulthood. Lennon et al. (2018) evaluated patient-reported functional mobility and life satisfaction in a cross-sectional study of young adults with CP. Interestingly, the FMS demonstrated stable functional mobility from childhood to adulthood. They also found that self-recall of childhood functional mobility using the FMS correlated highly in individuals who had received treatment for flexed knee gait as a child.

In our large cohort of children with CP, we found that even children with mild knee flexion contracture (5–14°) used a wheelchair for 46–61% of their daily mobility.

To our knowledge, the association between knee flexion contracture and functional mobility has not been explored before. It is important to note that the FMS classification and the assessment of standing and transfer ability are scored on how the individual actually moves in daily life, not necessarily what he or she can achieve at peak performance. This could be of interest to follow into adulthood, as it may reflect the continuous support and help these individuals need to receive to maintain active functional mobility in the community.

### Limitations

Several examiners (physiotherapists) with varying experience performed the measurement of knee flexion contracture. Measurement errors for goniometric assessments in children with CP have previously been reported, and no reliability study was performed in our study, which is a limitation (Stuberg et al. 1988). Nonetheless, the CPUP follow-up program strongly emphasizes practicing and learning how to measure passive range of motion with a goniometer in a standardized way (Nordmark et al. 2009). Furthermore, the variability of the measurements would most likely only influence the results marginally in this large cohort, which was also discussed in a study by Nordmark et al. (2009). Another limitation could be that the FMS classification is determined by the child and parents and therefore might be both underestimated and overestimated; however, the FMS classification system has proven reliable (Graham et al. 2004).

### Conclusion

Knee flexion contracture is common in children with CP and both mild and severe knee flexion contracture impacts functional mobility at all GMFCS levels. It is therefore important to prevent, detect, and treat knee flexion contracture.
